# *Pneumocystis jirovecii* colonization and its association with pulmonary diseases: a multicenter study based on a modified loop-mediated isothermal amplification assay

**DOI:** 10.1186/s12890-020-1111-4

**Published:** 2020-03-20

**Authors:** Ting Xue, Zhuang Ma, Fan Liu, Weiqin Du, Li He, Jinyan Wang, Chunli An

**Affiliations:** 1 Department of Microbiology and Parasitology, College of Basic Medical Science, China Medical University, 77 Puhe Road, Shenyang, People’s Republic of China; 2 Department of Respiratory and Critical Care Medicine, First Hospital of Shanxi Medical University, Taiyuan, People’s Republic of China; 3 Department of Respiratory Medicine, General Hospital of Shenyang Military Command, Shenyang, People’s Republic of China; 4 Department of Respiratory Internal Medicine, the First Affiliated Hospital of China Medical University, Shenyang, People’s Republic of China; 5 Department of Medical Laboratory, the Eleventh Affiliated Hospital of Shanxi Medical University, Lvliang, People’s Republic of China; 6 Department of Immunology, College of Basic Medical Science, China Medical University, Shenyang, People’s Republic of China

**Keywords:** *P. jirovecii*, Colonization, Respiratory diseases, Loop-mediated isothermal amplification

## Abstract

**Background:**

*Pneumocystis jirovecii* (*P. jirovecii*) is an opportunistic fungal pathogen and the role of its colonization in pulmonary diseases has become a popular focus in recent years. The aim of this study was to develop a modified loop-mediated isothermal amplification (LAMP) assay for detection of *Pneumocystis jirovecii* (*P. jirovecii*) DNA amongst non-HIV patients with various pulmonary diseases and use it to examine the prevalence and assess the association of *P. jirovecii* colonization with clinical characteristics of these diseases.

**Methods:**

We modified the previously reported LAMP assay for *P. jirovecii* by adding real-time detection. This method was used to detect *P. jirovecii* colonization in pulmonary samples collected from 403 non-HIV patients with various pulmonary diseases enrolled from 5 hospitals in China. We determined the prevalence of *P. jirovecii* colonization in 7 types of pulmonary diseases and assessed the association of *P. jirovecii* colonization with clinical characteristics of these diseases.

**Results:**

The modified LAMP assay showed no cross-reactivity with other common pulmonary microbes and was 1000 times more sensitive than that of conventional PCR. Using the modified LAMP assay, we detected *P. jirovecii* colonization in 281 (69.7%) of the 403 patients enrolled. *P. jirovecii* colonization was more common in interstitial lung diseases than in chronic obstructive pulmonary disease (COPD) (84.6% vs 64.5%, *P* < 0.05). Patients with acute exacerbation of COPD had a higher prevalence of *P. jirovecii* colonization compared to patients with stabilized COPD (67.4% vs 43.3%, *P* < 0.05). *P. jirovecii* colonization was associated with decreased pulmonary function, increased levels of 1,3-β-D-glucan and C-reactive protein, and decreased levels of CD4+ T-cell counts (*P* < 0.05 for each). Approximately 70% of *P. jirovecii* colonized patients had confections with other fungi or bacteria.

**Conclusions:**

We developed a modified LAMP assay for detecting *P. jirovecii*. Our multi-center study of 403 patients supports that *P. jirovecii* colonization is a risk factor for the development of pulmonary diseases and highlights the need to further study the pathogenesis and transmission of *P. jirovecii* colonization in pulmonary diseases.

## Background

*Pneumocystis jirovecii* (*P. jirovecii*) is a unicellular parasitic fungus and an opportunistic pathogen which almost exclusively infects the lungs [1, 2]. *P. jirovecii* infection may lead to a fatal *P. jirovecii* pneumonia (PJP) in immunocompromised patients, particularly in those infected with human immunodeficiency virus (HIV). While, the incidence of PJP in HIV-infected patients has decreased dramatically, mainly due to the widespread use of combination antiretroviral therapy, there are growing reports of PJP cases amongst HIV-negative individuals [3–7]. In addition, asymptomatic *P. jirovecii* colonization is being increasingly identified in immunocompetent individuals [2, 8]. In recent years, the role of *P. jirovecii* colonization in pulmonary diseases has become a popular focus, especially in those with chronic obstructive pulmonary disease (COPD) and sudden infant death syndrome [5, 8–11]. These asymptomatic PJP individuals, carrying *P. jirovecii*, may act as an important reservoirs of nosocomial infections. Therefore, it is necessary to clarify the role of *P. jirovecii* colonization in the pathogenesis of pulmonary diseases.

Various molecular methods for detection of *P. jirovecii* have been described previously, including conventional polymerase chain reaction (PCR) and real-time quantitative PCR [12–15]. Loop-mediated isothermal amplification (LAMP), originally developed by Notomi T et al. [16], is a novel, highly sensitive, and specific method to diagnose infectious diseases. However, SYBR Green I, used as a conventional intercalating fluorescent dye, resulted in a certain degree of inhibition of LAMP reaction, leading to several errors in the diagnosis [17, 18]. Thus, the aims of this study were to develop a modified real-time fluorescence LAMP assay by adding real time, and SYTO 9.0, as the fluorescent dye, for *P. jirovecii* detection, in order to avoid inhibition for LAMP reaction by SYBR Green I, and then, use it to examine the prevalence of *P. jirovecii* colonization amongst the non-HIV patients with various pulmonary diseases, and assess the association of *P. jirovecii* colonization with clinical characteristics of these diseases.

## Materials and methods

### Patient enrollment

This retrospective study was approved by the Institutional Ethics Review Board, China Medical University (2013070). Written informed consent was obtained from all the patients involved in this study. We enrolled a total of 403 patients with various pulmonary diseases hospitalized between October 2016 and January 2018, from 5 hospitals in China, including the First Affiliated Hospital of China Medical University, the Fourth Affiliated Hospital of China Medical University, the Fifth Affiliated Hospital of China Medical University, the Eleventh Affiliated Hospital of Shanxi Medical University, and the General Hospital of Shenyang Military Command. Clinical background information of patients was collected through the Case File System, available in the participating hospitals.

The patients with different symptoms and signs of pulmonary diseases, with non-HIV infections, without the clinical evidence or history of PJP symptoms, and without the definite diagnosis of PJP were included in the study [14, 19]. Besides considering the predisposing factors for the diseases, the pulmonary diseases were diagnosed not only on the basis of the clinical manifestations of the respiratory tract diseases (such as cough, fever, expectoration, wheezing, chest pain, chest tightness, hemoptysis, and dyspnea), but also chest imaging tests, bronchoscopic examination, and laboratory test, primarily including COPD [including the stable stage and acute exacerbations (AECOPD)], acute exacerbations of chronic bronchitis (AECB), interstitial lung diseases (ILDs), bronchiectasis, bronchial asthma, invasive pulmonary aspergillosis (IPA), and type I respiratory failure. Various diseases, included in the study, were diagnosed as follows: i) COPD was diagnosed mainly by confirming the presence of persistent airflow limitation according to a post-bronchodilator FEV1/FVC <  70%, history of risk factors such as exposure to cigarette smoke, and the Global Strategy for the Diagnosis, Management, and Prevention of Chronic Obstructive Lung Disease 2017 Report: GOLD Executive Summary [20]. In addition, the assessment of AECOPD met the GOLD guidelines, the diagnostic criteria of AECOPD given by American College of Chest Physicians (2011), and the European Respiratory Society guidelines [21]; ii) ILDs were primarily diagnosed by high-resolution computed tomography (HRCT) depicting the radiological manifestations of fibrosis in ILDs and sometimes, by bronchoalveolar lavage for the diagnosis of some particular types of ILDs [22, 23]; iii) AECB was diagnosed by considering the patients with mild to moderate syndromes of COPD (the diagnosis of COPD as mentioned above), patients who developed the exacerbated clinical presentations with increased dyspnea, increased sputum volume, and increased sputum purulence, and meanwhile, excluding pneumonia and other lung diseases by chest radiography [24]; iv) bronchiectasis was primarily diagnosed by HRCT of the chest [25], the gold standard for confirming the bronchiectasis, and considering the underlying causes such as post-infectious [26]; v) bronchial asthma was diagnosed through a detailed clinical history, physical examination, lung function, and detection of allergens [27]; vi) IPA was diagnosed by demonstrating *Aspergillus* in the respiratory samples and other contributions by 1,3-β-D-glucan assay, clinical manifestation of lower respiratory tract infection, and CT clinical characteristic with the air-crescent sign, halo sign, cavitation lesion, or nodules infarction; and vii) type I respiratory failure was diagnosed by laboratory studies such as evaluating the level of hypoxemia and hypercapnia via arterial blood gas and oxygen saturation, imaging examination, and pulmonary function test [28].

### Respiratory tract specimens

Before patients received diagnosis, antibiotics, and other treatments, specimens including blood, bronchoalveolar lavage fluid (BALF), and sputum samples were collected. Venous blood was collected in vacutainer tubes with different anticoagulants or coagulants and used immediately for the following tests: CD4+ T-cell counts (BD FACS Canto, USA), erythrocyte sedimentation rate (ESR) (Vital Monitor-20, Italy), and serum biochemical parameters including lactate dehydrogenase (LDH) (ADVIA 2400, SIEMENS Healthineers, Germany), pro-calcitonin (PCT) (Cobas 8000, Roche Diagnostics GmbH, Germany), C-reactive protein (CRP) (BN ProSpec System, SIEMENS Healthineers, Germany), and 1,3-β-D-glucan (BDG) (Goldstream MB-80, Era Biology Technology Co. Ltd. Tianjin, China). Pulmonary function (Medisoft S.A. Hyp’ Air, Belgium) and high-resolution computed tomography (HRCT) (BN ProSpec System, SIEMENS Healthineers, Germany) were measured by the following standard protocols in clinical practice. Each patient underwent HIV-1 and 2 antibody testing using the anti-HIV-1 and 2 antibody enzyme-linked immunosorbent assay (ELISA) diagnostic kit (Intec Products, Inc. Xiamen, China) to determine HIV infection status.

Amongst 403 patients, 324 patients provided sputum samples and 79 patients provided BALF samples. As the nature of the study is retrospective and severity status of the pulmonary disease was not available in the Case File System, we can only assume that patients who provided BALF sample had more severe disease status, as compared to patients who provide sputum samples. Moreover, patients providing sputum samples were not eligible for the BAL procedure. To collect the sputum samples, patients gargled with saline solution approximately three times to prevent oral microbial contamination before coughing up their morning sputa from the deep respiratory tract. Sputa were then collected in sterile containers and sent to clinical laboratories for bacterial and/or fungal culture and identification, respectively using the VITEC-2 system (ATB system, BioMérieux, Marcy-l’Étoile, France) and the Sensititre™, Aris 2X system (Thermo Fisher Scientific, USA). The remaining of the sputum samples were used to exact DNA and make slide smear to identify *P. jirovecii* microscopically following GMS and Giemsa staining.

Bronchoscopic examination was performed in 79 patients. BALF was collected using strict aseptic technique and filtered through a 2-layered nylon gauze to remove the mucus. The filtered BALF was divided for DNA extraction (described below) and slide smear staining by GMS and Giemsa staining.

### DNA extraction

BALF samples were centrifuged at 1500 rpm for 15 min and cell pellets were collected. The sputa were pretreated with 4% NaOH (w/v %), washed with saline solution approximately three times, and then centrifuged at 12,000 rpm for 10 min. After centrifugation, the supernatant was discarded. All cell pellets were washed three times with PBS and then subjected to DNA extraction following the traditional protocol involving proteinase K digestion, phenol-chloroform extraction, and ethanol precipitation. The DNA extracts were quantitated using the NanoDrop UV-Vis spectrophotometer (Thermo Fisher Scientific, USA) and stored at − 80 °C before use.

### Real-time fluorescence LAMP assay

All primers used in this study, including the external primer set (F3-B3), internal primer set (FIP-BIP), and loop primer set (LF-LB) have been described previously [5]. The LAMP reaction mixture contained 1.6 μM FIP-BIP, 0.2 μM F3-B3, 0.4 μM Loop F-B, and 2.5 μL 10× Isothermal Amplification Buffer including 0.1% Triton X-100 or 100 μg/ml BSA. The preferred buffer was 50 mM KCl, 10 mM Tris-HCl (pH 7.5), 0.1 mM EDTA, 1 mM DTT, 0.1% Triton X-100, and 50% glycerol (New England Biolabs, USA), 3.5 μL 10 mM dNTPs (Takara Biotechnology Co., LTD., Dalian, China), 1 μl 8 U/μl Bst DNA Polymerase, Large Fragment (New England Biolabs, USA), 1 μL100 mM MgSO_4_ (New England Biolabs, USA), 0.5 μL 10 μM SYTO 9.0 (Thermo Fisher Scientific, USA), 2 μL 5.0 M Betaine (Sigma-Aldrich, USA), 2 μL DNA template solution, and 6.5 μL RNase-free water (Takara Biotechnology Co., LTD., Dalian, China) up to a final volume of 25 μL. Each experiment included a no template control (with distilled water). The LAMP reaction was performed in the ABI 7500 system (ThermoFisher Scientific, USA) at 63 °C for 60 min, and terminated at 80 °C for 10 min.

The entire LAMP amplification process was monitored in real time and the fluorescent signals recorded automatically by the ABI 7500 system. Compared with the conventional LAMP, the real-time LAMP was analyzed by the amplification curves. At the end of the reaction, white precipitates formed at the bottom of the test tube. In the analysis of conventional LAMP, visual detection was observed by adding 2 μL 1000× SYBR Green I (Thermo Fisher Scientific, USA) and the detection of LAMP amplicons were evaluated by 2% agarose gel electrophoresis and stained with GoodView (SBS Genetech Co., Ltd.) for visualization under UV radiation.

### Conventional PCR

PCR was performed using the external primers targeting the 18S rRNA gene of *P. jirovecii* described elsewhere [5]. PCR mixture contained 12.5 μL of DNA polymerase Premix Taq™ (Takara Biotechnology Co., LTD., Dalian, China), 1–3 μL of DNA samples, 1 μL of 10 μM F3-B3 primer pair, and RNase-free water up to a final volume of 25 μL. Ultrapure distilled water was used as a negative control. PCR was performed under the following conditions: 94 °C for 5 min; 30 cycles of 94 °C for 30 s, 55 °C for 30 s, 72 °C for 30 s, and 72 °C for 8 min. PCR products were separated by electrophoresis on 1.5% agarose gels, stained with GoodView (SBS Genetech Co., LTD. Beijing, China), and visualized by UV radiation.

### Sensitivity and specificity of conventional PCR and the real-time fluorescence LAMP assay

To prepare a plasmid DNA standard, the PCR product, amplified using the external primers of the *P. jirovecii* 18S rRNA gene described above, was excised from the gel and purified using the agarose gel DNA extraction kit (Tiangen Biotech Co., LTD., Beijing, China). The recovered fragment was cloned into the TA cloning vector pMD18-T (Takara, Biotechnology Co., LTD., Dalian, China) with a standard TA cloning technique. DNA was extracted from one recombinant plasmid clone containing the correct sequence. DNA concentration was measured using the NanoDrop UV-Vis spectrophotometer (Thermo Fisher Scientific, USA). The sensitivities of the LAMP and PCR methods were compared using 10-fold serial dilutions of recombinant plasmid DNA (from 1 × 10^0^ copies/μL to 1 × 10^8^ copies/μL) in triplicate.

The specificities of the real-time fluorescence LAMP assay and conventional PCR were determined using genomic DNA from 10 other common respiratory microorganisms, including *Candida albicans, C. tropicalis, C. krusei, C. glabrata, C. parapsilosis, Aspergillus niger, Streptococcus pneumoniae, Escherichia coli, Acinetobacter baumannii,* and *Methicillin resistant Staphylococcus aureus* (MRSA)*.* The genomic DNA of each microorganism was extracted using the Plant Genomic DNA Kit (Tiangen Biotech Co., LTD., Beijing, China) or the TIANamp Bacteria DNA Kit (Tiangen Biotech Co., LTD., Beijing, China). An aliquot of 10–100 ng of genomic DNA from each microorganism was used in each reaction.

### Statistical analysis

All data were analyzed using the statistical analysis software SPSS (version 20.0, IBM Corporation, Chicago, IL, USA). Counting variables are represented as frequencies or percentages. Continuous variables are represented as the mean ± SD. Data related to clinical findings, imaging examinations, serologic parameters, and other indicators were compared using the χ^2^ test. Fisher’s exact test was used for the clinical data analysis when the number of samples was less than 40 or the expected value was less than 1. *P* values less than 0.05 were considered as statistically significant.

## Results

### Development of a modified real-time fluorescence LAMP assay for *P. jirovecii* detection

**Fig. 1:**
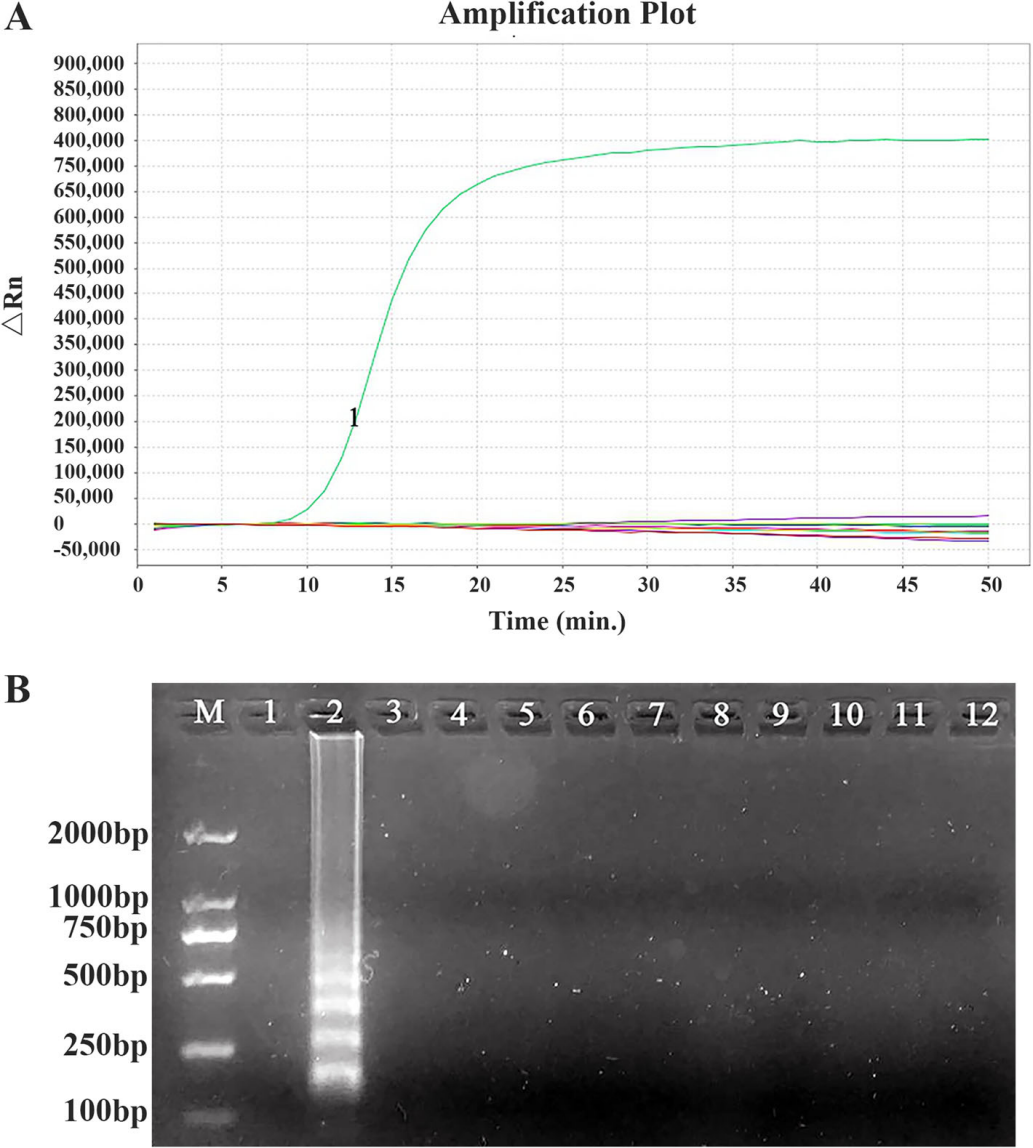
The specificity of the real-time fluorescence LAMP assay for *P. jirovecii* detection. **a** A representative result of the LAMP assay. The green curve (labeled as 1) represents the LAMP amplification plot of one positive specimen of *P. jirovecii*. Other flat curves near the bottom represent amplification plots of control samples, including the negative control (with water) and other common respiratory microbes such as *Candida albicans, C. tropicalis, C. krusei, C. glabrata, C. parapsilosis, Aspergillus niger, Streptococcus pneumoniae, Escherichia coli, Acinetobacter baumannii,* and *Methicillin resistant Staphylococcus aureus* (MRSA). **b** A representative result of 2% agarose gel electrophoresis analysis of LAMP products. M, DNA size marker; lane 1, no template control; lane 2, *P. jirovecii-*positive specimen. Lanes 312 are *C. albicans*, *C. tropicalis*, *C. krusei*, *C. glabrata*, *C. parapsilosis*, *A. nige*, *S. pneumoniae*, *E. coli*, *A. baumannii*, and *Methicillin resistant S. aureus* (MRSA), respectively

**Fig. 2:**
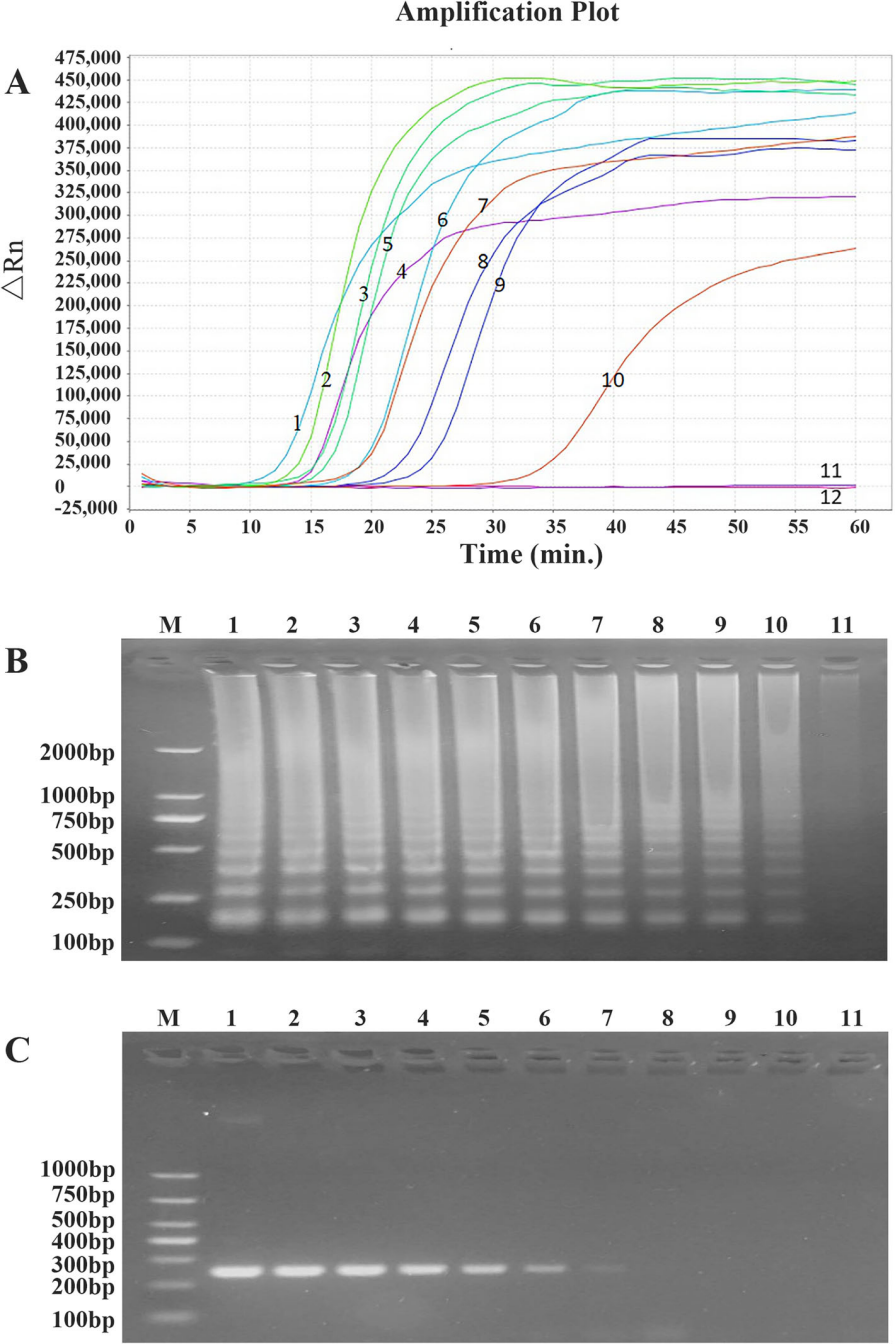
Comparison of the sensitivity of real-time fluorescence LAMP assay and conventional PCR using 10-fold serial dilutions of recombinant plasmid DNA containing *P. jirovecii* 18S rRNA gene. **a** Amplification plots of the real-time fluorescence LAMP assay (from 1 × 10^10^ copies/μl for curve 1 to for 1 × 10^0^ copies/μl curve 11, and no template control for curve 12). **b** 2% agarose gel electrophoresis of LAMP products from serial dilutions of plasmid from 1 × 10^10^ copies/μl for lane 1 to for 1 × 10^0^ copies/μl lane 11. M, DNA size marker. **c** 2% gel electrophoresis of conversional PCR products from serial dilutions of plasmid from 1 × 10^10^ copies/μl for lane 1 to for 1 × 10^0^ copies/μl lane 11. M, DNA size marker

We developed a modified real-time fluorescence LAMP assay of *P. jirovecii.* This assay showed no cross-reactivity with 10 other common respiratory pathogenic microorganisms tested, suggesting its high specificity (Fig. 1). The sensitivity of this assay was compared with that of conventional PCR using serial dilutions of recombinant plasmid DNA containing *P. jirovecii* 18S rRNA gene. As shown in Figs. 2, the lowest detection threshold was 1 × 10 copies/μl for the LAMP assay, which was 1000 times more sensitive than that of conventional PCR (1 × 10^4^ copies/μl).

**Fig. 3:**
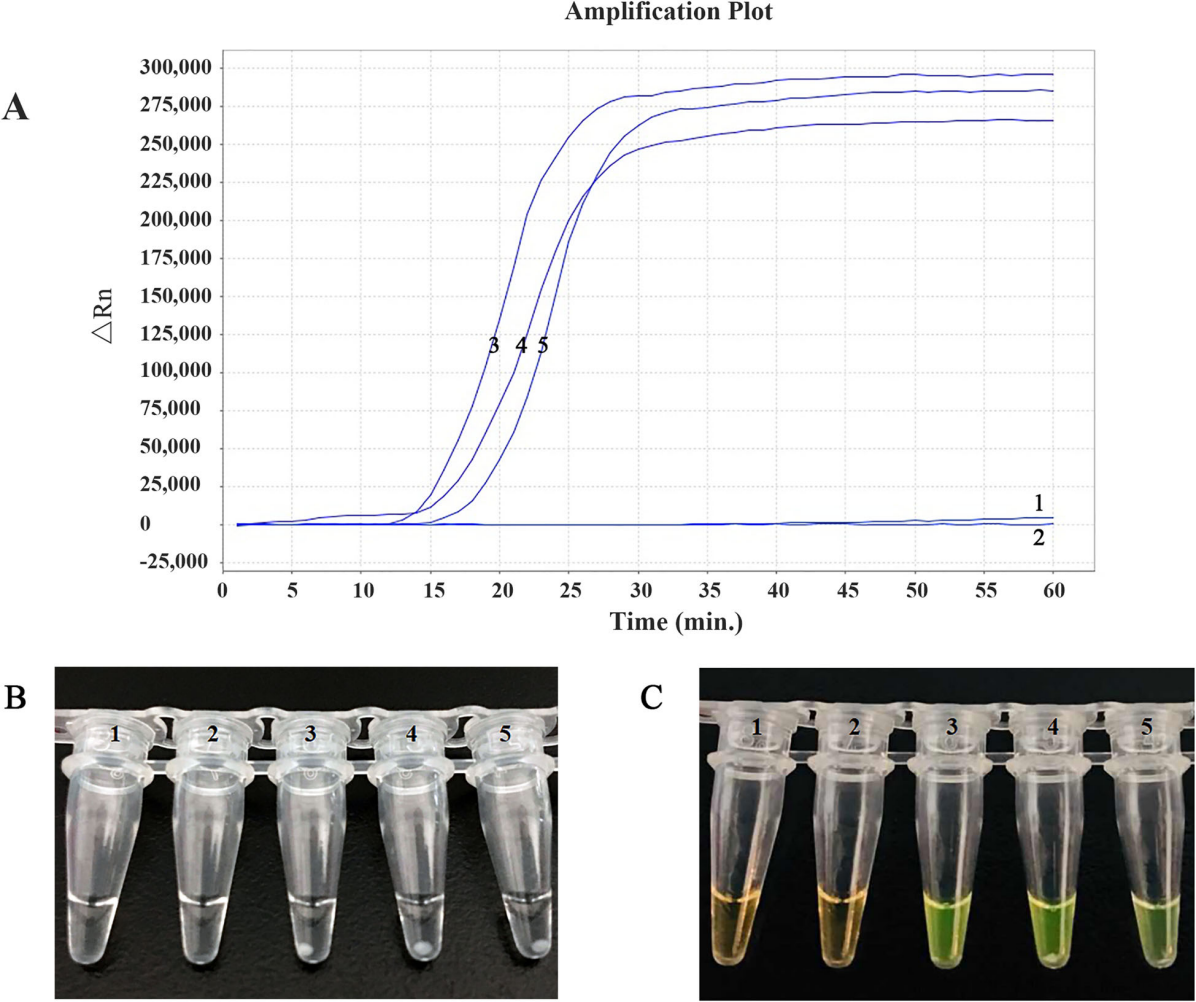
Comparison of different methods for detecting LAMP products. **a** Amplification plots of real-time fluorescence LAMP. Curve 1, no template control; curve 2, *P. jirovecii*-negative patient specimen; curves 3–5, *P. jirovecii*-positive specimens. **b** Direct visual detection of end-point products as white precipitate at the bottom of reaction tubes: Tube 1, no template control (no precipitate); tube 2, *P. jirovecii*-negative patient specimen (no precipitate); tubes 3–5, *P. jirovecii*-positive specimens (precipitate at the bottom). **c** Visual detection of end-point products mixed with SYBR Green I fluorescent dye (Thermo Fisher Scientific). Tube 1, No template control (orange color); tube 2, *P. jirovecii*-negative patient specimen (orange); tubes 3–5, *P. jirovecii*-positive patient specimens (emerald green)

We also compared the sensitivity of this modified LAMP assay with conventional PCR using clinical specimens of patients with various respiratory diseases. The positive rates of *P. jirovecii* were 69.7% (281/403) by the LAMP assay (Fig. 3) and 40.5% (163/403) by conventional PCR. In only one case, positive by both assays, cyst forms of *P. jirovecii* were detected by GMS and Giemsa staining. There was a good coincidence of the *P. jirovecii* detection rate between the LAMP and conventional PCR. All the positive specimens detected by conventional PCR were also positive in LAMP, however, the all positive specimens detected by LAMP were not necessarily positive in conventional PCR. All these data indicate that our modified real-time fluorescence LAMP method is reliable for the detection of *P. jirovecii*.

### Prevalence of *P. jirovecii* colonization in sputa and BALF from patients with various pulmonary diseases

Of the 403 patients enrolled, the mean age was 66.7 ± 12.5 years, including 248 (61.5%) males and 155 (38.5%) females. The overall prevalence of *P. jirovecii* colonization amongst all enrolled patients was 69.7% (281/403) (Table 1). The prevalence of *P. jirovecii* colonization in BALF of patients was higher compared with it in sputa, but the comparative differences between them were not significant (75.9% vs 68.2%, *P* >  0.05) (Table 2). The prevalence of *P. jirovecii* colonization in ILDs patients was significantly higher compared with COPD and AECOPD patients (85% vs 67 and 43.3%, *P* < 0.01). Furthermore, the prevalence of *P. jirovecii* colonization in AECOPD patients was significantly higher than those with stabilized COPD (67.4% vs 43.3%, *P* < 0.01) (Table 1).

**Table 1:** *P. jirovecii* colonization rate in patients with pulmonary diseases

	Patients with colonization (*n* = 281)	Patients without colonization (*n* = 122)	All patients (*n* = 403)	Positive rate (%)
Age (years)	66.21 ± 13.23	67.98 ± 10.63	66.74 ± 12.51	
Male (%)	66.5	33.5	61.5	
COPD	160	88	248	64.52^*^
AECOPD	147	71	218	67.43^**^
Stable stage	13	17	30	43.33
Interstitial lung diseases	55	10	65	84.62
IIP	47	8	55	84.45
Sarcoidosis	8	0	8	100
PAP	0	2	2	0
AECB	27	13	40	67.50
Bronchiectasis	30	8	38	78.95
Bronchial asthma	2	3	5	40.0
IPA	4	0	4	100
Type 1 respiratory failure	3	0	3	100

Abbreviations: *COPD* chronic obstructive pulmonary disease, *AECOPD* acute exacerbation of COPD, *AECB* acute exacerbations of chronic bronchitis, *IPA* invasive pulmonary aspergillosis, *PAP* pulmonary alveolar proteinosis, *IIP* idiopathic interstitial pneumonia

Data are expressed as numbers (%). ^*^P < 0.01 compared with ILDs; *P* > 0.05 compared with AECB, bronchiectasis and bronchial asthma; ** *P* < 0.01 compared with the stable stage

**Table 2:** *P. jirovecii* colonization rate in BALF and sputa of the patients

	Patients with colonization (*n* = 281)	Patients without colonization (*n* = 122)	Total (*n* = 403)	Positive rate (%)
*P. jirovecii* colonization in BALF	60	19	79	75.94^*^
*P. jirovecii* colonization in sputa	221	103	324	68.20

^*^P > 0.05 compared with *P. jirovecii* colonization in sputa

### Presence of various comorbidities in patients with *P. jirovecii* colonization and pulmonary diseases

More than half (230 or 57%) of all the enrolled patients presented with coexisting diseases including autoimmune diseases, connective tissue diseases, and other diseases (Table 3). Majority of these patients with comorbidities were positive for *P. jirovecii* (167 vs 63). There were 24 patients with autoimmune diseases including rheumatoid arthritis (RA), autoimmune thyroid disease (AITD), chronic glomerulonephritis (CGN), Sjogren’s syndrome, connective tissue disease (CTD), and mixed connective tissue diseases (MCTD). Notably, the frequency of *P. jirovecii* detection in patients with autoimmune diseases was higher than those with the hypertension, diabetes, and coronary heart disease. Moreover, particular finding worth mentioning is the presence of a statistically significant difference in the frequency of *P. jirovecii* gene detection between patients with autoimmune diseases and those with the cardiac insufficiency (*P* < 0.05).

**Table 3:** Comorbidities in patients with *P. jirovecii* colonization

Comorbidities	Patients with colonization	Patients without colonization	Total	Positive rate (%)
Hypertension	45	12	57	78.95
Cardiac insufficiency	31	20	51	60.78
Diabetes mellitus	25	8	33	75.76
Coronary heart disease	24	9	33	72.73
Autoimmune diseases	24	5	29	82.75^**#**^
Systemic inflammatory response syndrome	9	2	11	81.82
Others	9	7	16	56.25
*Total*	167	63	230	72.61

^**#**^*P* > 0.05 compared with hypertension, diabetes, coronary heart disease and systemic inflammatory response syndrome; *P* < 0.05, > 0.01, compared with the cardiac insufficiency group

### Concurrent infection with other fungi or bacteria in patients with *P. jirovecii* colonization

Most of the patients with *P. jirovecii* colonization (71/102 or 69.6%) had concurrent infection with other fungi and bacteria (Table 4). There were no significant differences in pair-wise comparison of the *P. jirovecii* colonization rates amongst patients co-infected with bacteria, those with other fungi, and those with both other fungi and bacteria (66.2% vs 69.6% vs 85.7%, *P* >  0.05). Furthermore, comparison between AECOPD patients co-infected with bacteria or fungi and COPD patients in stable stage co-infected with bacteria or fungi showed that there was no significant difference (25.69% vs 33.33%, *P* >  0.05) (Table 5).

**Table 4:** Concurrent infection with other fungi and bacteria in patients with *P. jirovecii* colonization

Microorganism	Positive	Negative	Total	Positive rate (%)
Bacteria	43	22	65	66.15^*^
*Streptococcus pneumoniae*	10	5	15	66.67
*Pseudomonas aeruginosa*	11	3	14	78.57
*Klebsiella pneumoniae*	5	4	9	55.56
*Haemophilus influenzae*	4	3	7	57.14
*Acinetobacter baumannii*	4	1	5	80.00
*Others*	9	6	15	60.00
Other fungi	16	7	23	69.57
*Candida albicans*	11	6	17	64.71
*Aspergillus fumigatus*	4	0	4	100
*Others*	1	1	2	50.00
Other fungi and bacteria	12	2	14	85.71
*P. aeruginosa and C. albicans*	3	0	3	100
*Others*	9	2	11	81.82
Total	71	31	102	69.61

^*^P > 0.05 compared with fungal infection alone and infection with both fungi bacteria

**Table 5:** Concurrent infection with other fungi or bacteria in patients with COPD

	Patients with concurrent infection	Patients without concurrent infection	Total	Positive rate (%)
AECOPD	56	162	218	25.69^*^
COPD Stable stage	10	20	30	33.33
Total	66	182	248	36.26

^*^P > 0.05 compared with concurrent infection with fungi or bacteria among COPD patients in stable stage

### Association of *P. jirovecii* colonization with clinical characteristics of patients with various pulmonary diseases

As illustrated in Table 6, no significant differences in PCT, LDH, and ESR measurements were observed between patients with and without *P. jirovecii* colonization (*P* >  0.05). However, patients with *P. jirovecii* colonization showed significantly increased levels of BDG and CRP, and significantly decreased levels of pulmonary function and CD4+ T-cell counts compared to patients without *P. jirovecii* colonization (*P* < 0.05). In addition, compared to patients without *P. jirovecii* colonization, patients with *P. jirovecii* colonization had significantly more abnormal findings on thoracic HRCT imaging, including reticulation opacities, cystic lesions, septal thickening, nodules, irregular linear opacities, and consolidation (*P* < 0.05).

**Table 6:** Association of *P. jirovecii* colonization with laboratory findings of the patients

	Positive	Negative	Total	Positive rate (%)
Serum BDG levels				
Positive (> 10 pg/ml)	34	5	39	81.18^a^
Negative (< 10 pg/ml)	128	50	178	71.91
Serum PCT levels				
Positive (> 0.5 ng/mL)	59	22	81	72.84^b^
Negative (< 0.5 ng/mL)	103	33	136	75.74
Serum LDH levels				
Positive (> 225 U/L)	84	33	117	71.79^c^
Negative (< 225 U/L)	197	89	286	68.88
Serum CRP levels				
Positive (> 5 mg/L)	171	59	230	74.35^d^
Negative (< 5 mg/L)	110	63	173	63.58
ESR test				
Abnormal	78	33	111	70.27^e^
Normal	109	51	160	68.13
Thoracic HRCT findings				
GGO positive	67	14	81	82.72^f^
GGO negative	95	41	136	69.85
Pulmonary function				
FEV1/FVC positive (< 70%)	25	6	31	80.65^g^
FEV1/FVC negative (> 70%)	14	11	25	56.00
CD_4_^+^ T-cell count				
< 410/mm^3^	23	2	25	92.00^h^
> 410/mm^3^	53	24	77	68.83

Abbreviations: *BDG* 1,3-β-D-glucan, *PCT* pro-calcitonin, *LDH* lactate dehydrogenase, *CRP* C-reactive protein, *ESR* erythrocyte sedimentation rate, *GGO* ground-glass opacity, *HRCT* high-resolution computed tomography, *FEV1* forced expiratory volume in one second, *FVC* forced vital capacity

^a^*P* < 0.05, > 0.01, Compared with serum BDG < 10 pg/mL

^b^*P* > 0.05 Compared with serum PCT < 0.5 ng/mL

^c^*P* > 0.05 compared with LDH levels < 225 U/L

^d^*P* < 0.05, > 0.01, compared with CRP negative (< 5 mg/L)

^e^*P* > 0.05 compared with ESR normal. ESR abnormal (> 15 mm/h for men and > 20 mm/h for women). ESR normal (≤ 15 mm/h for men and ≤ 20 mm/h for women)

^f^*P* < 0.05, > 0.01, compared with GGO negative

^g^*P* < 0.05, > 0.01, compared with FEV1/FVC negative (> 70%)

^h^*P* < 0.05, > 0.01, compared with CD4+ T-cell count > 410/mm^3^

## Discussion

*P. jirovecii* is a life-threatening opportunistic pathogen in immunocompromised patients. As extensively reviewed by Morris and Norris, *Pneumocystis* colonization, in contrast to *Pneumocystis* infection, is defined as the presence of *Pneumocystis* organisms (usually identified by PCR-based molecular biological techniques) without signs or symptoms of acute pneumonia [2, 8]. Colonization with *P. jirovecii* can even occur in individuals with normal immunity due to the development of highly efficient strategies to escape both innate and acquired immune defenses in host [1, 2]. These colonized individuals may serve as reservoirs of nosocomial infections. A high-level prevalence of *P. jirovecii* colonization in immunocompetent individuals has been reported in numerous studies throughout the world [2, 29]. The role of *P. jirovecii* colonization in certain pulmonary diseases, particularly amongst patients with COPD, has gained increasing attention [5, 8, 9, 30]. In the present multicenter study involving 403 non-HIV patients with various pulmonary diseases, we found a high prevalence of *P. jirovecii* colonization and this was associated with some clinical and laboratory characteristics of patients with pulmonary diseases, supporting the involvement of *P. jirovecii* colonization in pulmonary diseases.

A similar study, LAMP assays for the detection of *P. jirovecii* gene amongst the patients with chronic pulmonary diseases, was previously performed in our hospital in 2015 [5]. SYBR Green I, a fluorescent dye, had been used previously in this assay. However, SYBR Green I, used as the conventional intercalating fluorescent dye, had several drawbacks, including significant inhibition, despite its widespread use for LAMP reaction and real-time PCR [17, 18]. SYTO 9.0 has been confirmed as having the least inhibitory effect amongst the fluorescent intercalating dyes tested [17]. In order to avoid inhibition of the LAMP reaction due to SYBR Green I, we first developed a modified real-time fluorescence LAMP assay by using SYTO 9.0 (Thermo Fisher Scientific), as the fluorescent dye for *P. jirovecii*. Compared with the conventional PCR, the modified LAMP assay showed 1000 times more sensitivity, whereas the LAMP assay reported previously was 200 times more sensitive. We found that the lowest detection threshold for this modified assay was lower than that reported in the previous study using indirect comparison (10 copies vs 50 copies per reaction), which indicated that the sensitivity of detection has been modified. So, in this study, SYTO 9.0 fluorescent dye, added to the reaction of modified real-time fluorescence LAMP assay mixture, demonstrated higher amplification efficiency and sensitivity, without detectable inhibitory effect [17]. To better assess the prevalence of *P. jirovecii* colonization, we used this modified real-time fluorescence LAMP assay for the detection of *P. jirovecii*. The overall frequency of colonization by *P. jirovecii* (69.7%), observed in this study, appears to be higher than that reported in previous studies (16–63.3%) [5, 31]. Moreover, in this study, the prevalence of *P. jirovecii* colonization in BALF of patients (75.9%) is higher than that reported previously (21/30 or 70%) [30]. In addition, Nested PCR (mt LSU rRNA as *Pneumocystis* target gene) was compared with real-time fluorescence LAMP, and the positive rate was 58.56% (236/403), which was lower than the positive rate of real-time fluorescence LAMP used in this study. The reasons for these observed differences are unclear. It may be related to the superiorities or advantages of this modified LAMP mentioned above, in addition, also related to different target genes, different detection methods, different sample processing, different underlying diseases and diagnosis, or different patient population tested.

The high prevalence of *P. jirovecii* colonization observed in this study is consistent with elevated serum beta-glucan levels in patients with *P. jirovecii* colonization compared to patients without *P. jirovecii* colonization (Table 6), as has been reported in various studies [32–34]. In addition to the high prevalence of *P. jirovecii* colonization in various pulmonary diseases, this study provides several other lines of evidence supporting *P. jirovecii* colonization as a risk factor for the development of pulmonary diseases. In this study, HRCT imaging findings suggested that the ground-glass opacity (GGO) could be a result of *P. jirovecii* colonization in patients with pulmonary diseases. Compared to patients without *P. jirovecii* colonization, patients with *P. jirovecii* colonization showed a decreased pulmonary function as assessed by the FEV1/FVC values (Table 6), which may suggest a possibility of damage to the respiratory tract due to long-term colonization. This possibility is further supported by the elevated serum C-reactive protein (CRP) levels in patients with *P. jirovecii* colonization, as CRP is a sensitive marker of inflammation and tissue damage. Finally, consistent with the previous studies [35–37], we also observed decreased CD4+ T-cell counts in patients with *P. jirovecii* colonization compared to patients without *P. jirovecii* colonization, which suggests an impaired T cell-mediated immunity.

Patients with chronic pulmonary diseases frequently suffer from various comorbidities (Table 3), which may also contribute to the severity of diseases. In this study, we found a high rate of underlying autoimmune diseases in patients with *P. jirovecii* colonization (Table 3). This finding can be explained by the widespread administration of systemic high-dose steroids in these patients [38, 39].

Despite increasing reports of *P. jirovecii* colonization, there are only a few studies of coinfection with other respiratory pathogens in patients with *P. jirovecii* colonization [40–42]. To study whether the presence of other microorganisms in lungs facilitate or inhibit the *P. jirovecii* colonization, the concurrent infection with other fungi and bacteria in patients with *P. jirovecii* colonization were investigated. And, we found that the majority of patients with *P. jirovecii* colonization had concurrent infections with other fungal or bacterial pathogens (Table 4). Although the presence of other organisms might be the most common reason for the association with exacerbation in COPD, in this study, there was no significant difference in comparison of the prevalence of concurrent infection with other organisms in AECOPD patients and those with stabilized COPD (Table 5). In addition, PCT is a sensitive biomarker of bacterial infection. However, no significant differences in PCT measurements were observed between patients with and without *P. jirovecii* colonization. This indirectly indicates that *P. jirovecii* colonization might contribute to the risk of COPD exacerbations and other various pulmonary diseases. Further studies are needed to assess the differential contribution of these pathogens to the pulmonary diseases.

This study did have certain limitations. First, a direct comparison between the LAMP assay previously reported and the modified assay established in this study had not been performed yet. Further analysis should be performed after the direct comparison of the assays in the future clinical studies. Second, there were certain heterogeneities in the sample types (sputum and BALF samples) and underlying clinical conditions, which might have caused biases in data interpretation. Third, we did not subtract coinfection with other fungi or bacteria when assessing the association of *P. jirovecii* colonization with clinical characteristics due to the small sample size after subtraction. Fourth, sputum specimens are not the best specimens to examine the prevalence of coinfection with other fungi or bacteria in the lungs, due to unavoidable contamination with normal microbial flora in the oral cavity and nasopharynx. In this study, we used a large number of sputum specimens, due to the limited availability of BALF samples. Lastly, we represented the data on the basis of individual pulmonary diseases; however, due to retrospective nature of this study, we lacked the data on severity of individual pulmonary disease and thus, could not represent the data on the basis of severity, such as the GOLD staging for COPD and the severity classification of asthma.

## Conclusions

In summary, we have developed a modified LAMP assay for detecting *P. jirovecii* colonization. This multi-center study involving 403 patients, using this assay, has provided additional new evidence for the involvement of *P. jirovecii* colonization in the development of pulmonary diseases in non-HIV patients, and highlights the need to further study the pathogenesis and transmission of *P. jirovecii* in pulmonary diseases.

## Data Availability

The data used in this study are available from the corresponding author on reasonable request.

## Abbreviations

PJP: *Pneumocystis jirovecii* pneumonia
COPD: Chronic obstructive pulmonary disease
HIV: Human immunodeficiency virus
LAMP: Loop-mediated isothermal amplification
AECOPD: Acute exacerbations of chronic obstructive pulmonary disease
AECB: Acute exacerbations of chronic bronchitis
ILDs: Interstitial lung diseases
IPA: Invasive pulmonary aspergillosis
BALF: Bronchoalveolar lavage fluid
ESR: Erythrocyte sedimentation rate
LDH: Lactate dehydrogenase
PCT: Pro-calcitonin
CRP: C-reactive protein
BDG: 1,3-β-D-glucan
HRCT: High-resolution computed tomography
GGO: The ground-glass opacity
